# Treatment protocols for growth hormone-secreting pituitary adenomas combined with craniofacial fibrous dysplasia: A case report of atypical McCune-Albright syndrome

**DOI:** 10.3892/etm.2014.1792

**Published:** 2014-06-19

**Authors:** JIA XU, XI LI, CHANG-SHENG LV, YING CHEN, MENG WANG, JIAN-FENG LIU, LAI GUI

**Affiliations:** Department of Maxillofacial Surgery, Plastic Surgery Hospital, Chinese Academy of Medical Science, Peking Union Medical College, Beijing 100144, P.R. China

**Keywords:** McCune-Albright syndrome, fibrous dysplasia, growth hormone-secreting pituitary adenoma

## Abstract

McCune-Albright syndrome (MAS) is a rare, post-zygotic (non-germline) disorder, characterized by hypersecretory endocrinopathies, fibrous dysplasia of the bone and café-au-lait macules. The most common endocrine dysfunction is gonadal hyperfunction; thus, hypersecretion of growth hormones (GHs) as a manifestation of endocrine hyperfunction in MAS is rarely reported. MAS affects both genders, although the majority of cases have been reported in young females. Atypical presentations of MAS, with only one or two of the classic symptoms, have been previously described, but remain particularly challenging due to the lack of a diagnostic phenotype. In patients with atypical MAS, analysis of mutations in the gene of the α-subunit of the stimulatory G-protein is limited; thus, diagnosis is based on clinical judgment. In the present study, a male with polyostotic fibrous dysplasia and GH-secreting pituitary adenomas, diagnosed with atypical MAS, was reported. The pituitary adenoma was effectively treated with radiotherapy and the patient underwent surgery for the polyostotic fibrous dysplasia, with marked improvements observed in appearance.

## Introduction

McCune-Albright syndrome (MAS) is a rare, sporadic condition with an estimated prevalence between 1/100,000 and 1/1,000,000 (1), which is characterized by the following three features: Fibrous dysplasia (usually polyostotic), café-au-lait macules and endocrine hyperfunction (2). Endocrinopathies often include sexual precocity, as well as hyperthyroidism, hypercortisolism, growth hormone (GH) excess and hyperprolactinemia. MAS is caused by post-zygotic mutations of G-proteins, such as GNAS (1,3–5). Successful diagnosis using GNAS mutation analysis has been reported to be associated with disease severity, yet <50% yield positive results in patients with the classic triad. However, atypical presentations of MAS, with only one or two of the classic symptoms, have been reported in literature and remain particularly challenging due to the lack of a diagnostic phenotype. Therefore, the utility of GNAS mutation analysis in these patients is limited and diagnosis is often based on clinical judgment (6).

In classic and atypical forms, hypersecretion of GHs is not often associated with MAS (7). Symptoms are caused by excessive GH-secreting pituitary adenoma and craniofacial fibrous dysplasia; thus, the overall aim of treatment is to inhibit excessive GH secretion, with optimal facial esthetics following treatment. The most effective treatment of atypical MAS with GH-secreting pituitary adenoma is surgical resection, however, the treatment protocols are varied depending on the complexity of the symptoms. Therefore, treatment of MAS remains a challenge for clinicians. In the present study, a case of atypical MAS associated with GH-secreting pituitary adenoma was reported, with effective management using radiotherapy and surgery.

## Case report

A 27-year-old male patient was admitted to the Department of Maxillofacial Surgery (Peking Union Medical College, Beijing, China) with frontal deformity. Written informed consent was obtained from the patient. At the age of six years, the patient started to present with abnormal progressive growth of the cranial bones, primarily in the left frontal bone, and a subsequent progressive decline in vision in the left eye. At 12-years of age, the patient lost vision in the left eye and was diagnosed at a different hospital with ‘skull structural abnormalities’, but received no treatment. The patient’s feet then began to grow rapidly and at the age of 13 years, the height of the patient increased to 190 cm. At 21-years of age (height, 198.3 cm), the patient was admitted to Peking Union Medical Hospital and was diagnosed with a pituitary adenoma, for which the patient received radiotherapy for 26 days. The patient stopped growing and had no recurrence for five years. To improve appearance, the patient sought treatment at the Peking Union Medical Hospital. The patient had no family history of this disorder.

**Figure 1:**
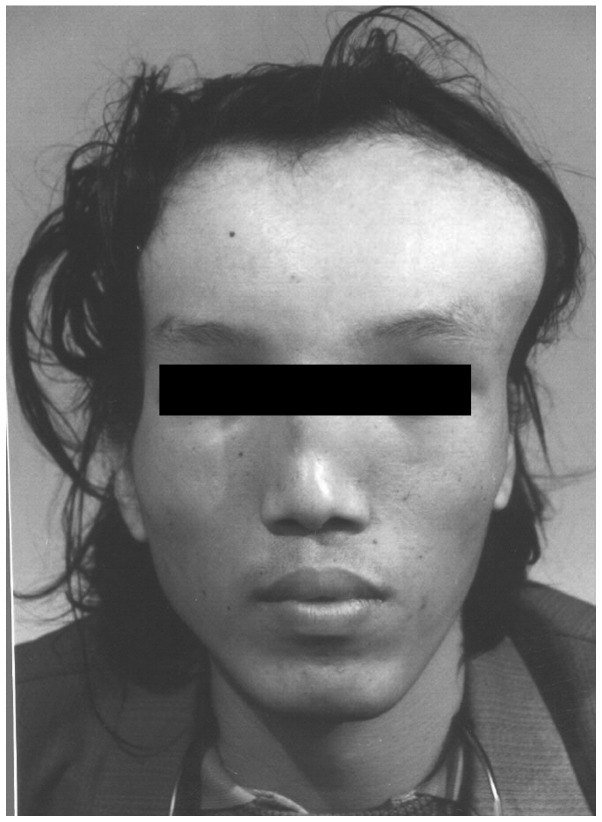
Anterior aspect of the patient pre-surgery.

**Figure 2:**
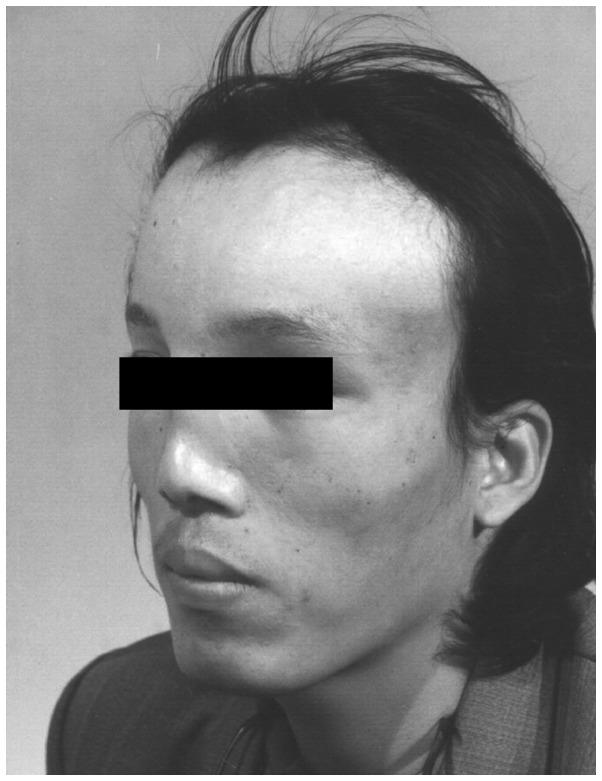
Lateral aspect of the patient pre-surgery.

**Figure 3:**
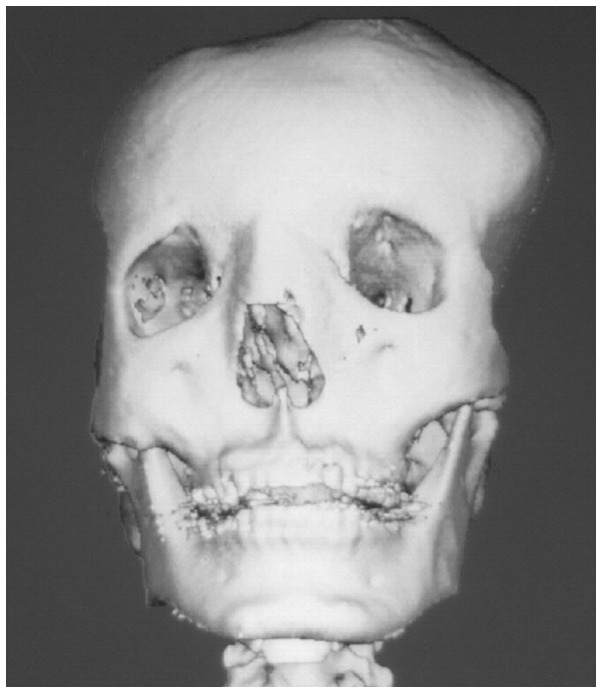
Three-dimensional computed tomography reconstruction demonstrating the anterior observations of the craniofacial dysplasia.

**Figure 4:**
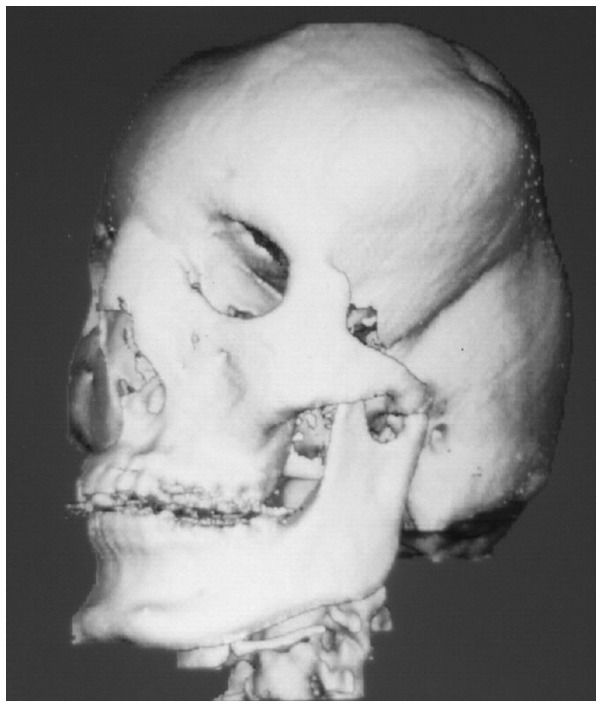
Three-dimensional computed tomography reconstruction demonstrating the lateral observations of the craniofacial dysplasia.

**Figure 5:**
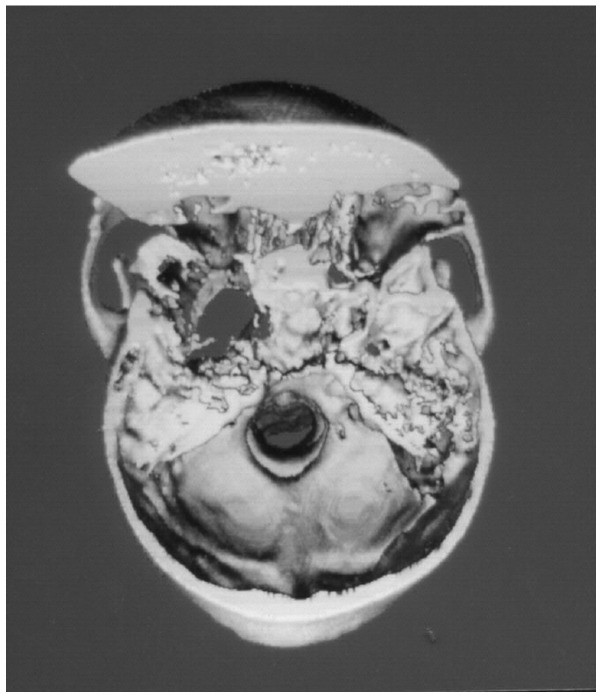
Three-dimensional computed tomography reconstruction demonstrating the skull base observations of the craniofacial dysplasia.

**Figure 6:**
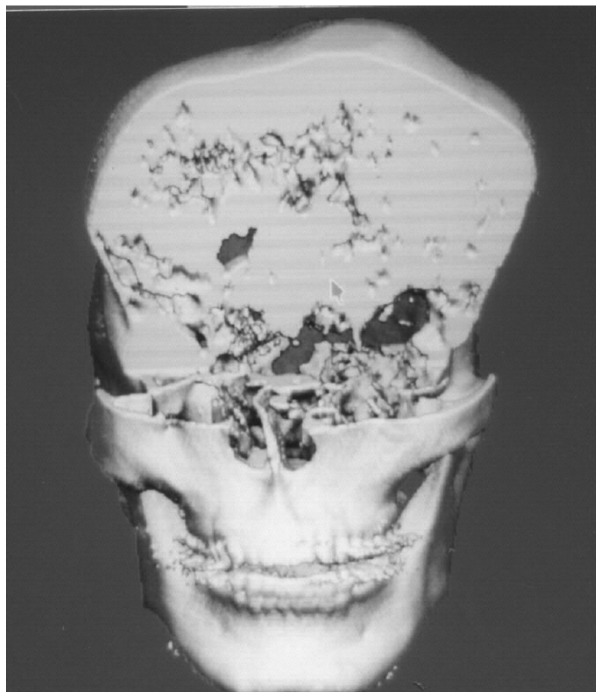
Three-dimensional computed tomography reconstruction demonstrating the interior observations of the craniofacial dysplasia.

On examination, the patient appeared anxious and had a pulse rate of 100/min, blood pressure of 140/90 mmHg and a height of 199.1 cm. Examination revealed an abnormally broad and prominent forehead (Fig. 1), which was more marked on the left side (Fig. 2). The bone near the sagittal suture was abnormally prominent; however, no café-au-lait macules were found on the surface of the whole body. Computed tomography three-dimensional reconstruction revealed the involvement of the frontal, temporal and parietal bones, as well as the orbit and extensive skull base (Fig. 3–6). Since the polyostotic fibrous dysplasia was associated with the pituitary adenoma, a diagnosis of atypical MAS was considered.

A conservative shaving approach was selected for the treatment of the deformity. Sections of the lesions were resected using an osteotome, and the bones were then contoured with a high-speed burr for esthetic reasons. A biopsy of the resected bone was consistent with fibrous dysplasia, with areas of fibrosis and woven bone.

**Figure 7:**
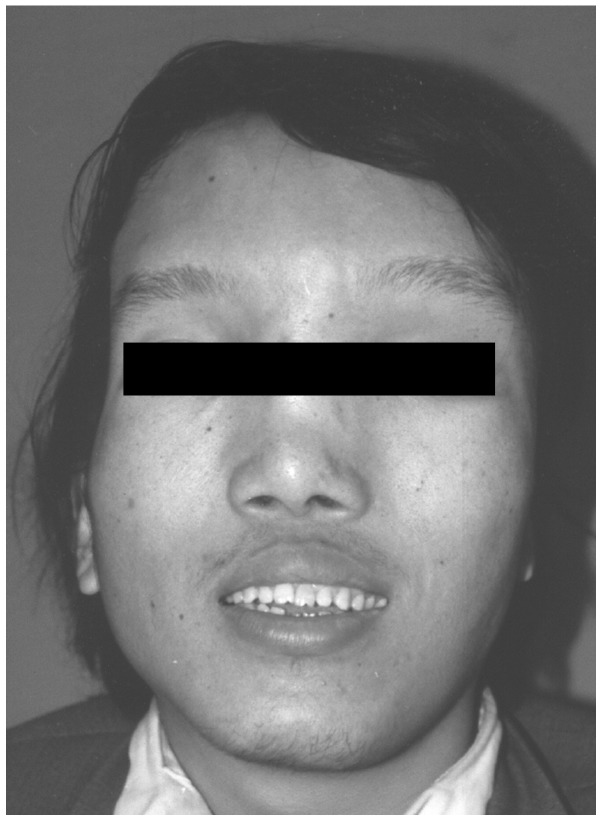
Anterior aspect of the patient post-surgery.

**Figure 8:**
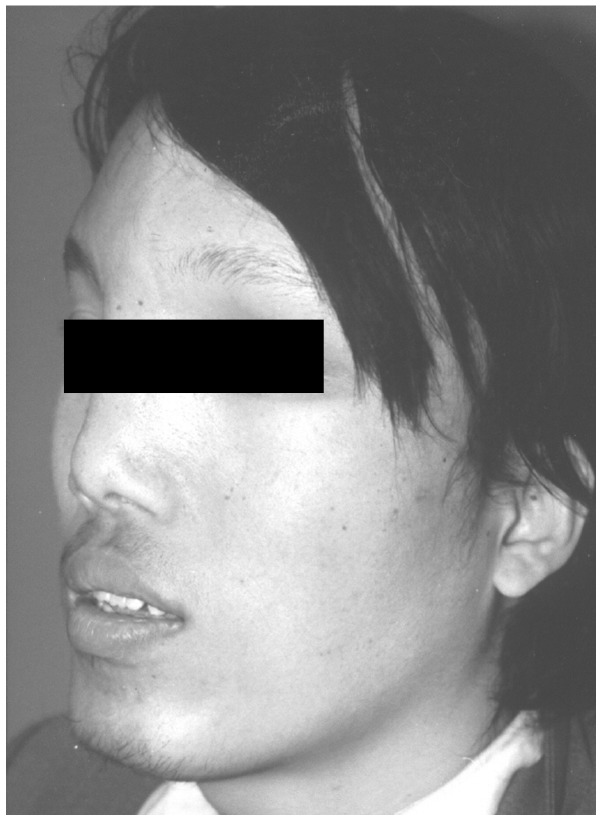
Lateral aspect of the patient post-surgery.

The postoperative course was uneventful, and the patient was satisfied with the appearance (Fig. 7 and 8). There was no evidence of postoperative dysplastic recurrence during the one-year follow-up.

## Discussion

MAS is a rare disorder, thus, is often underdiagnosed and may be overlooked in patients with fibrous dysplasia (8), particularly in patients with atypical MAS. In patients with classic MAS, excessive secretion of GHs may accelerate fibrous dysplasia, particularly craniofacial fibrous dysplasia (3), resulting in visual and auditory dysfunction and macrocephaly (9). Therefore, the treatment of GH-secreting pituitary adenoma is the first step in MAS treatment, which remains to be a challenge for clinicians.

Surgery, radiotherapy and medication may be used for the treatment of MAS; however, effective treatment protocols of GH-secreting pituitary adenoma may be difficult to select due to the complexity of MAS. The optimal current treatment for pituitary adenoma, with the exception of prolactinomas, is surgical resection. However, in patients with MAS, even subtotal pituitary adenoma resection is often prohibited by severe skull base fibrous dysplasia (10). Radiation therapy remains controversial in patients with MAS due to the risk of sarcomatous transformation of the fibrous dysplasia lesions within the radiation portals (11,12). However, in a previous study investigating fibrous dysplasia at the Mayo Clinic, the results supported the use of radiotherapy (13). The study demonstrated that while 13 of the 1,122 patients with fibrous dysplasia (mostly non-MAS patients) developed sarcomatous bone transformation within the radiation portals, an almost equal number of patients did so with (n=13) and without (n=14) previous radiation to the region. However, to the best of our knowledge, no previous studies have specifically addressed the use of radiosurgery in the treatment of pituitary adenomas in patients with MAS; thus, radiosurgery may be a viable option. In the present case study, due to the severe skull base fibrous dysplasia, the patient received effective radiotherapy for the pituitary adenoma, with no malignant transformation observed through radiation imaging examination recently.

Surgery is the primary treatment option for craniofacial fibrous dysplasia. The surgery varies according to the different situations, including the degree of cranial nerve involvement, the severity of the symptoms and the cosmetic demands of the patient. Generally, the aim of treatment is to correct or prevent functional problems and to achieve a normal facial appearance (14–16). With regard to the management of polyostotic fibrous dysplasia, a mixed approach may be considered, using conservative shaving or radical resection. In the present case, a conservative shaving approach was used for the following reasons. Firstly, as the lesion was benign and the dysplasia involved the skull base, the risks of complete resection were very difficultly weighed; thus, conservative shaving or osseous contouring was recommended. Secondly, the patient refused radical excision for esthetic reasons and hoped for minimal surgical risks. Finally, although the recurrence rate following contouring has been reported to be as high as 25% (17), the patient was beyond adolescence and the tumor had not grown for five years, reaching a static phase; therefore this approach was selected for the patient.

In conclusion, the present study reported a case of a 27-year-old male with MAS combined with GH-secreting pituitary adenoma. The patient was successfully treated with radiosurgery and a conservative shaving approach. The patient continues to undergo follow-up observations by the methods of photography and radiation imaging examination to assess any recurrent and malignant transformation in the remaining lesion region.
